# Raman Spectroscopy characterization extracellular vesicles from bovine placenta and peripheral blood mononuclear cells

**DOI:** 10.1371/journal.pone.0235214

**Published:** 2020-07-02

**Authors:** Han Zhang, Ana Caroline Silva, Wei Zhang, Heloisa Rutigliano, Anhong Zhou

**Affiliations:** 1 Department of Biological Engineering, Utah State University, Logan, Utah, United States of America; 2 Department of Animal Dairy and Veterinary Sciences, Utah State University, Logan, Utah, United States of America; Tianjin University, CHINA

## Abstract

Placenta-derived extracellular vesicles (EVs) are involved in communication between the placenta and maternal immune cells possibly leading to a modulation of maternal T-cell signaling components. The ability to identify EVs in maternal blood may lead to the development of diagnostic and treatment tools for pregnancy complications. The objective of this work was to differentiate EVs from bovine placenta (trophoblast) and peripheral blood mononuclear cells (PBMC) by a label-free, non-invasive Raman spectroscopy technique. Extracellular vesicles were isolated by ultracentrifugation. Dynamic light scattering (DLS) and scanning electron microscopy (SEM) were applied to verify the presence and the size distribution of EVs. Raman peaks at 728 cm^-1^ (collagen) and 1573 cm^-1^ (protein) were observed only in PBMC-derived EVs, while the peaks 702 cm^-1^ (cholesterol) and 1553 cm^-1^ (amide) appeared only in trophoblast-derived EVs. The discrimination of the Raman spectral fingerprints for both types of EVs from different animals was performed by principal component analysis (PCA) and linear discriminant analysis (LDA). The PCA and LDA results clearly segregated the spectral clusters between the two types of EVs. Moreover, the PBMC-derived EVs from different animals were indistinguishable, while the trophoblast-derived EVs from three placental samples of different gestational ages showed separate clusters. This study reports for the first time the Raman characteristic peaks for identification of PBMC and trophoblast-derived EVs. The development of this method also provides a potential tool for further studies investigating the causes and potential treatments for pregnancy complications.

## 1 Introduction

Extracellular vesicles (EVs) are phospholipid membrane complexes that contain proteins, lipids, and nucleic acids. They are classified into three main categories: exosomes (40–120 nm), microvesicles (50–1000 nm) and apoptotic bodies (500–4,000 nm). Exosomes are formed when a late endosomal vesicle (multivesicular body) fuses with the plasma membrane and releases its vesicles in the extracellular space. As a result of their unique biogenesis, exosomes are enriched with unique miRNAs and proteins such as the cluster of differentiation (CD) 63, CD9, CD81.[1] Microvesicles result from the budding of the plasma membrane and its formation is stimulated by translocation of phosphatidylserine to the outer membrane leaflet. Their formation is completed through actin-myosin interactions that promote contraction of cytoskeletal structures. Apoptotic bodies are distinguished by the presence of organelles within them. They are formed due to membrane blebbing induced by signaling proteins involved with programmed cell death.[2] Extracellular vesicles mediate intercellular communication within and between tissues and organs.[3] In mouse and humans, pregnancy-associated exosomes have been shown to mediate intercellular communication between the placenta and the maternal immune cells. Placenta-derived exosomes have been found to suppress maternal T-cell signaling components possibly leading to immunomodulation and maternal immune tolerance to fetal antigens.[4, 5]

Reproductive efficiency of dairy cattle has reduced over the years. While the causes of this decrease are still unclear, it is known that embryonic loss is a major contributor.[6] Therefore, understanding the mechanisms involved in achieving a successful pregnancy may lead to the development of new early diagnostic methods of pregnancy complications, and strategies to prevent embryonic loss and improve pregnancy rates in livestock species of great economic relevance such as dairy cows. Increasing our understanding of how placental EVs communicate with the maternal endometrium and immune system has the potential to identify biomarkers and/or therapeutic targets for pregnancy-associated disorders.[7]

A better understanding of how trophoblast-derived EVs communicate with the immune system can help comprehend several pregnancy complications, such as early embryonic loss and preeclampsia.[8] Working with livestock species presents limitations related to protein and extracellular vesicle analyses for research and diagnostic purposes. The most commonly used strategies for EV analysis are the identification and characterization of their nucleic acid, protein and lipid content through extensive genomic, proteomic and lipidomic approaches. For example, one of the most straightforward ways of characterizing EVs is to determine their protein composition using immunoblotting assays and immunosorbent EV assays. [9] However, these methods are costly, time-consuming, and require a large amount of highly concentrated EV samples. Especially, the detection of protein markers in bovine samples is a large challenge due to the limited availability of commercial antibodies which compromises the ability to identify EVs in maternal blood. Moreover, EVs are difficult to characterize due to their nanoscale size and the heterogeneity of their origin and composition. There is no gold standard technique to analyse, isolate or determine the precise physical, biochemical, and bio-molecular characteristics of EV populations. Thus, there is a need for simpler and faster methods to analyse the molecular components of EVs.

Although many alternative optical methods have been used to analyse exosomes, most of them provide only limited biochemical information. [10] For example, fluorophore-assisted methods such as fluorescence microscopy (FM),[11] fluorescence correlation microscopy (FCM),[12] and stimulated emission depletion microscopy (SEDM)[13] provide the biochemical information of only targeted biological components in the exosome. Whereas, other scattering techniques such as dynamic light scattering (DLS),[14] nanoparticle tracking analysis (NTA)[15] and scattering flow cytometry (SFC)[16] only present limited physical information such as size distribution of the exosomes. Atomic force microscopy (AFM) can be performed without requiring any sample labeling. It can measure mechanical properties such as elasticity and stiffness of vesicles. [9] However, low throughput and need of specific skills are a major limitation for the use of this technique.

Raman spectroscopy was reported as a non-invasive technique able to discriminate EVs derived from different tissues.[17–20] [21, 22] Raman spectroscopy is a label-free technique based on inelastic scattering of laser light due to the interaction of photons with molecular vibrations. The Raman spectrum of inelastically scattered photons provides information about the biochemical components of the sample. Moreover, compared with conventional methods, Raman spectroscopy is also non-destructive, and the analysis of each sample typically needs less than 1 minute depending on the scan range. Raman spectra of each type of EV act as a fingerprint, which is significant to identify the tissue/cell source of EVs. In this study, Raman spectroscopy and principal component analysis (PCA) were applied to test the hypothesis that bovine placental EVs and peripheral blood mononuclear cell (PBMC)-derived EVs have different cargos. We aimed to determine biochemical differences between these EVs to aid in future functional studies. This study provides the first Raman-based characterization of bovine EVs derived from placental cells and PBMC. Our findings provide evidence that Raman spectroscopy followed by PCA analysis has the capability to differentiate between bovine EVs derived from these two cell types which indicate that the cargo of these EVs differ.

## 2 Materials and methods

### 2.1 Materials

Spinner minimum essential medium (SMEM) and DNAse type I were purchased from Sigma-Aldrich (St Louis, MO). Penicillin, streptomycin, fetal bovine serum (FBS), phosphate-buffered saline (PBS) and 0.5% trypsin–EDTA were purchased from Life Technologies (Carlsbad, CA). Magnesium Fluoride (MgF_2_) wafers were purchased from ALB Materials Inc. (Hendersion, NV).

### 2.2 Cell culture

The Institutional Animal Care and Use Committee at Utah State University approved the use of animal samples in this project (Animal Care Protocol USU IACUC 10035). Placental tissue was collected at a local abattoir. After collection, samples were immediately transported to the laboratory in 0.06% chlorhexidine and digested for 30 min with 0.25% of trypsin and 1500 IU/ml of DNAse type I (Sigma-Aldrich). The cell suspension was layered over a Percoll gradient (40%). The interfaces were harvested, washed in SMEM with 10% FBS. Isolated trophoblasts were seeded at a density of 1 x 10^6^ cells/mL in cell culture flasks. These cells were cultured in EV depleted culture medium (SMEM supplemented with penicillin 50 units/ml, streptomycin 50 μg/ml and 5% fetal bovine serum).

Peripheral blood mononuclear cells were isolated by density gradient centrifugation (Accu-Paque, Accurate Chem. & Sci. Corp). Isolated PBMC cells were seeded at a density of 1 x 10^6^ cells/mL in cell culture flasks. These cells were cultured in EV depleted culture medium (SMEM supplemented with penicillin 50 units/ml, streptomycin 50 μg/ml and 5% fetal bovine serum).

### 2.3 Extracellular vesicle isolation

**Fig 1:**
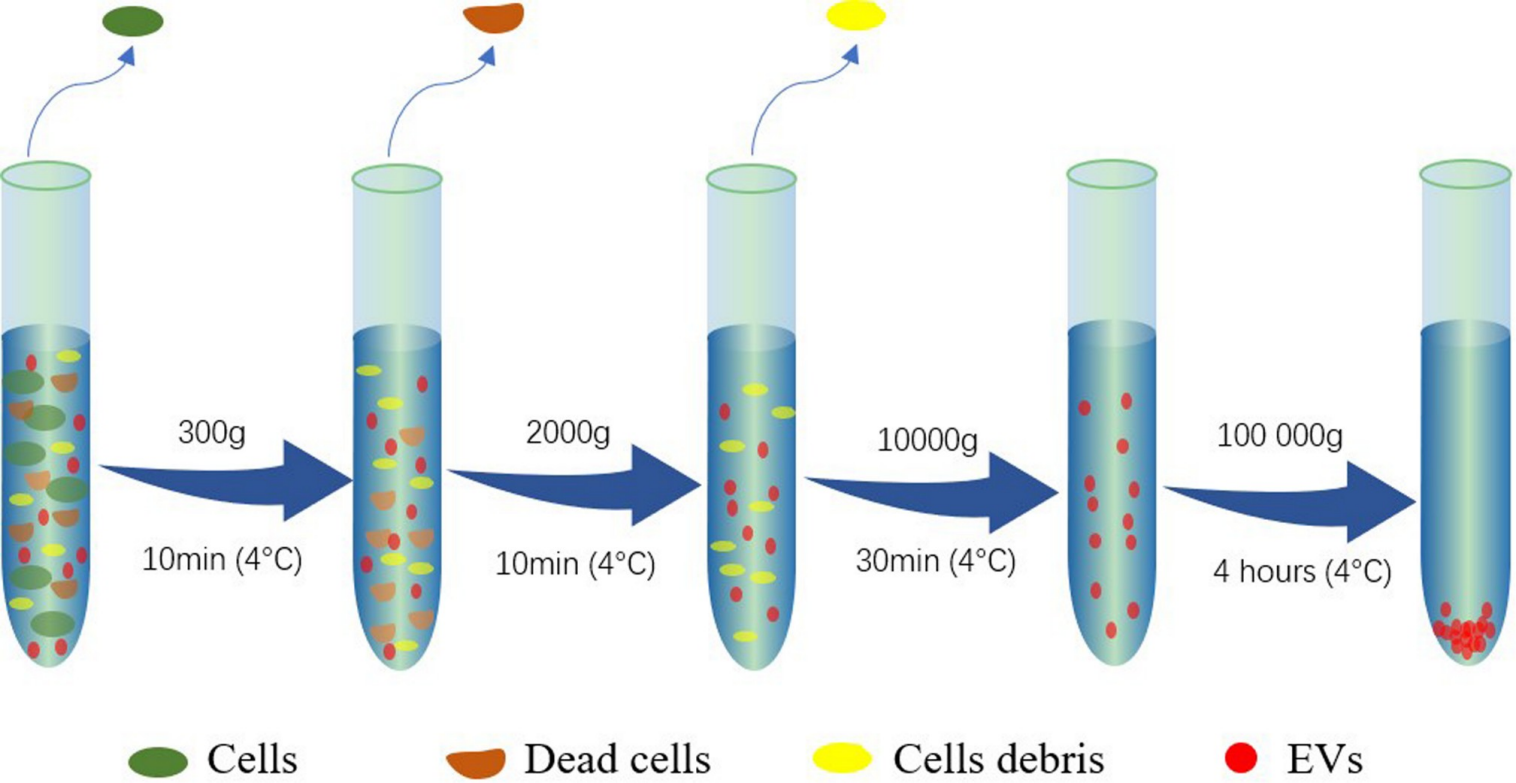
Multiple centrifugation steps for extracellular vesicle isolation.

After 48 hours of culture, cell culture supernatant was collected and EVs were isolated as described previously [23] with a modification that the ultracentrifugation time was increased to 4 hours (Fig 1). The pellets were suspended in 200μL of PBS. Trophoblast cells were collected from three different pregnant cows labeled as T001, T002 and T003 at 130, 162 and 207 days of gestation, respectively. Peripheral blood mononuclear cells were collected from another group of three non-pregnant cows and labeled P001, P002 and P003. The detail EV sample information of the screened cows is listed in S1 Table.

### 2.4 Assessment of EV Isolation by dynamic light scattering and SEM

Dynamic light scattering (DLS, DynaPro NanoStar, Wyatt Instrument Co.) was applied to determine the dimensional distribution of different types of EVs. Moreover, size distribution and concentration of the ultracentrifugation preparation was assessed by nanoparticle tracking analyses (NTA) using a NanoSight NS300 instrument (NanoSight Ltd, Amesbury, UK), the standard measurement protocol was applied followed by processing with software (NanoSight) to track visible particles. A sample of 50 μL of EVs diluted 1000x in PBS was transferred to a test tube and read by the NTA.

The morphology of the EVs was characterized by FEI Quanta FEG 650 Scanning Electron Microscopy (SEM). The EV pellet that formed after ultracentrifugation was suspended in 2% glutaraldehyde and incubated at 4°C overnight. This solution (17ml) was ultracentrifuged (100,000 x g) for 4h and the pellet was suspended in 200 μL of deionized water. A sample of the fixed EVs (20 μL) was transferred to a round MgF_2_ optical window and dried for 2h at 37°C. The samples were coated by gold (10 nm thickness) before SEM imaging. Samples were imaged under low vacuum mode at a magnification of 25,000 to 200,000 times.

### 2.5 Raman spectroscopy characterization

**Fig 2:**
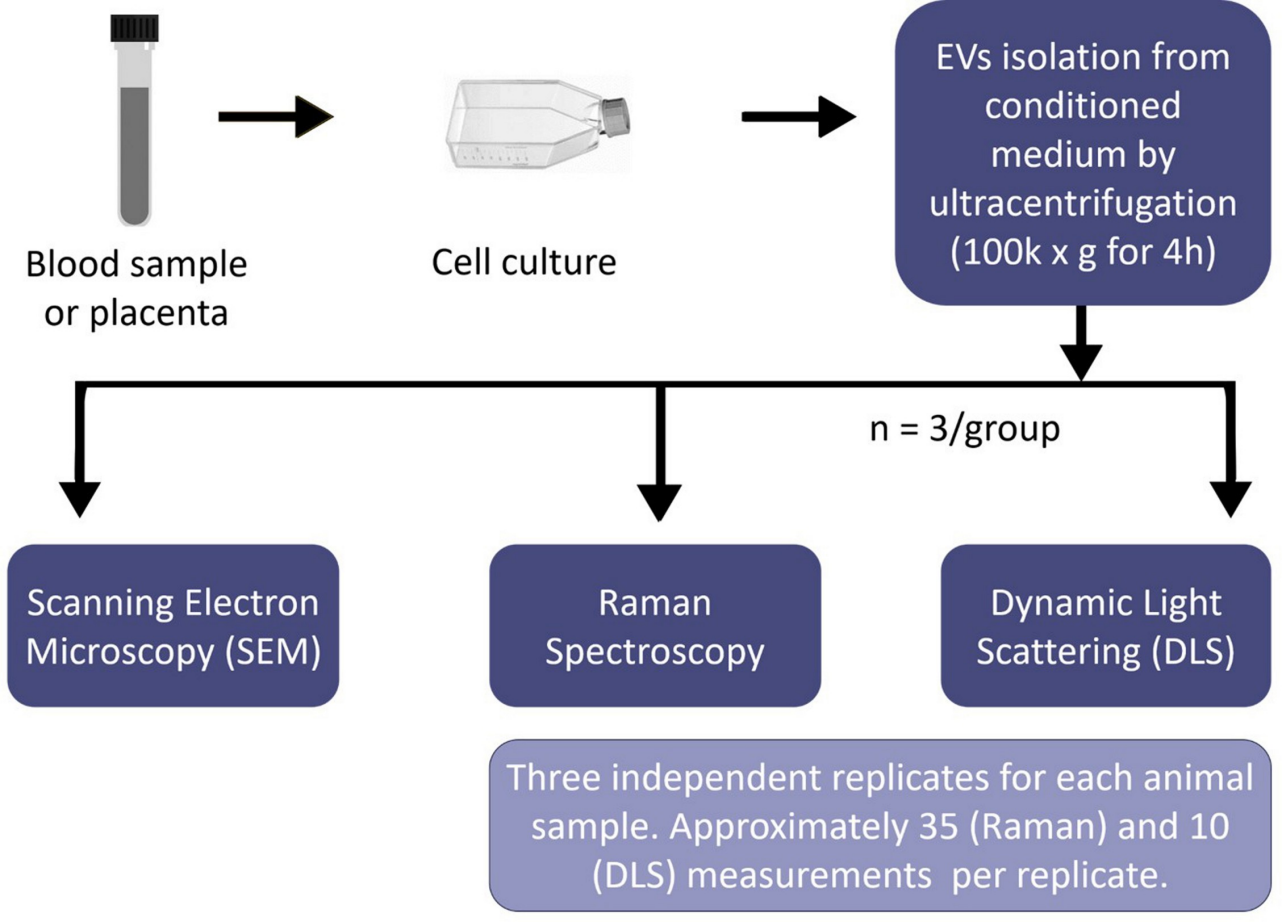
Steps of sample preparation to data collection.

The Renishaw inVia Raman spectrometer (Renishaw plc, UK) connected to a Leica microscope (Leica DMLM, Leica Microsystems, Buffalo Grove, IL, USA) was used for the cell spectra collection. A 785 nm near-IR laser was equipped for Raman spectrometer. The 50× dry lens objective in the spectral range 600 cm^-1^ to 1800 cm^-1^ was implemented for the spectra collection. Silicon wafer was used for calibration before data collection (adjusted to 520.5±0.1 cm^-1^ for silicon peak). The exposure time was 10 seconds for 1 accumulation at 100% laser power density for all the cell samples. Cosmic rays in raw spectra were removed using “zap” function in Renishaw Wire 3.4 software. A volume of 20 μL of EV sample was loaded on an MgF_2_ optical window and dried for 1h at 37°C before the spectra collection. For each EV type, spectra from three independent animals were collected (one optical window for each animal, total of 6 optical windows used). A total of 35 measurements were taken at each MgF_2_ optical window. Fig 2 illustrates the steps from sample preparation to data collection.

### 2.6 Data analysis

In this study, PCA was applied to examine the spectral differences between trophoblast and PBMC-derived EVs. The differences among animals collected within each tissue type were also assessed. The first ten PC scores were used in a supervised classification model, linear discriminant analysis (LDA), in order to discriminate and classify the data by maximizing the variance between groups. One-way Analysis of variance (ANOVA) was applied for analysing the difference of the characteristic spectral peaks among EVs. OriginPro 2018 (v. b9.5.1.195, OriginLab, Northampton, MA, USA) was used for data manipulation and analysis. The detailed data analysis method is described in S1 Data of supplemental materials.

## 3 Results and discussion

### 3.1 Extracellular vesicle isolation

**Fig 3:**
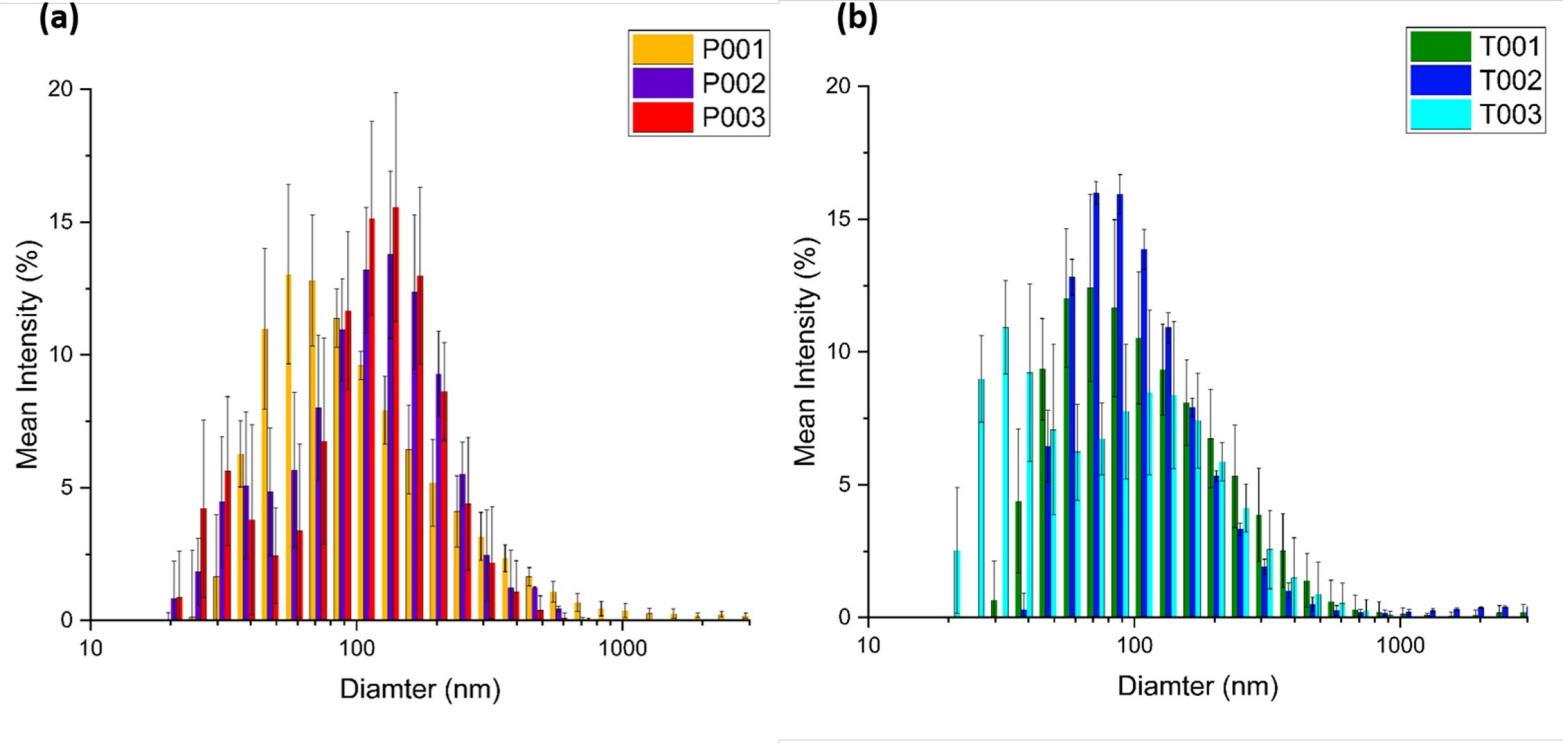
Size distribution by dynamic light scattering (DLS). (PBMC)-derived EVs (a) and trophoblast-derived EVs (b) from different cows *(N = 5)*.

The achievement of EV concentration plays an important role in obtaining excellent Raman signal. The precipitated pellet of EVs can be observed in the red circled area in S1 Fig. After fully resuspending the EVs in PBS, their size was measured by DLS. The results from DLS are illustrated in Fig 3. The charts show that, although the distribution of EV sizes varied between animals, exosomes (50 to 150 nm) were the most abundant type of EVs in conditioned media derived from trophoblast and PBMC culture. The average size of PBMC-derived EVs (102.3 nm±118.1 nm) was larger than that of trophoblast-derived EVs (89.7 nm±99.9 nm) based on a weighted average calculation. T-test was conducted using mean size distribution, The P value was 0.8112 indicating there was no significant difference in size between these two groups of EVs. Both groups of EVs are still in the same average size range of exosomes. The NTA analysis of our ultracentrifugation preparation found the mode diameter to be 83nm and the mean diameter to be 95± 38 nm for trophoblast derived EVs. The concentration of our preparation was about 3.23 x 10^11^ particles/ml.

### 3.2 Scanning electron microscopy-morphology

**Fig 4:**
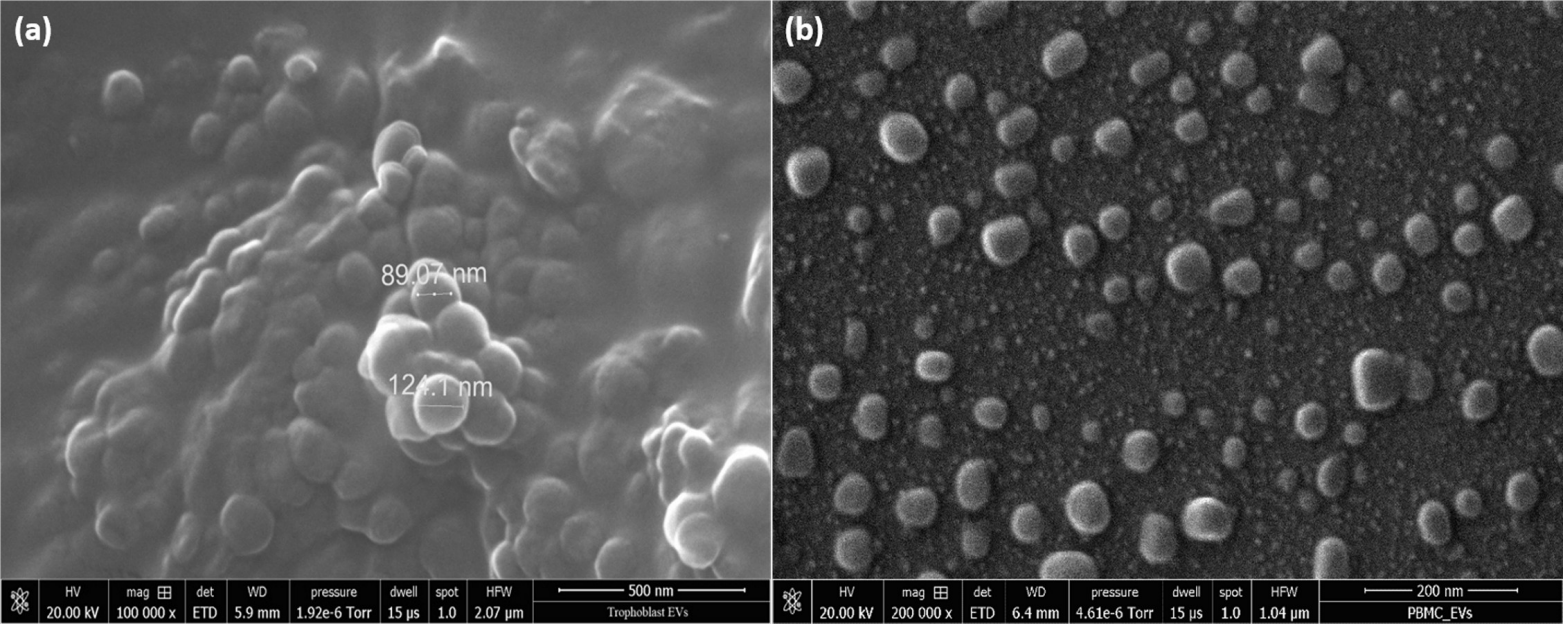
The scanning electron microscopy (SEM) morphology image of EVs after centrifugation isolation. Trophoblast-derived EVs (a) and PBMC derived EVs (b) at 100k magnification.

Scanning electron microscopy was employed to visually demonstrate the presence of EVs in the resuspended pellet. The SEM morphology is illustrated in Fig 4. In most cases, the EVs are seen as aggregate. Imaging single EVs was a challenge considering the small field of view. The major reason for the aggregation observed in Fig 4 could be the extremely long ultracentrifugation time or the use of glutaraldehyde primary fixation, since this chemical promotes protein cross-linking. According to Fig 4, the dimensional distribution of either trophoblast or PBMC-derived EVs were close to the results measured by DLS. The diameter of EVs varied from approximately 50 to 150 nm.(b)

### 3.3 Raman spectroscopy

In this study, biochemical fingerprints from trophoblast and PBMC-derived EVs were investigated by Raman spectroscopy. A volume of 20 μL of EV suspension was loaded on MgF_2_ slices and dried for 1 h at 37°C before spectra collection. Higher Raman counts were found at the edge area of the dried drips, which indicate that the EVs were concentrated on the edges of the MgF_2_ slices after drying. Because of this pattern, our spectra were all collected at randomly selected sites on the edges of all samples. The main reason why MgF_2_ was used as a substrate for Raman measurement of EV samples in this study is because it does not produce an intense background signal in the EV’s spectral fingerprint area, and it has an extremely wide transmission range. [24, 25] S2 Fig compares the Raman spectra of pure MgF_2_, dried PBS buffer on MgF_2_ and dried trophoblast-derived EVs on MgF_2_. According to the results, the Raman spectral intensity of MgF_2_ background is low, smooth, and negligible; and the average signal was approximately 350 counts. No peak was found between the regions 600–1800 cm^-1^. The dried PBS crystals on MgF_2_ elevated the baseline slightly by 100–200 counts. Since both MgF_2_ and PBS buffer have no noticeable peaks between 600–1800 cm^-1^, the effect of background can be eliminated or minimized after baseline subtraction.

**Fig 5:**
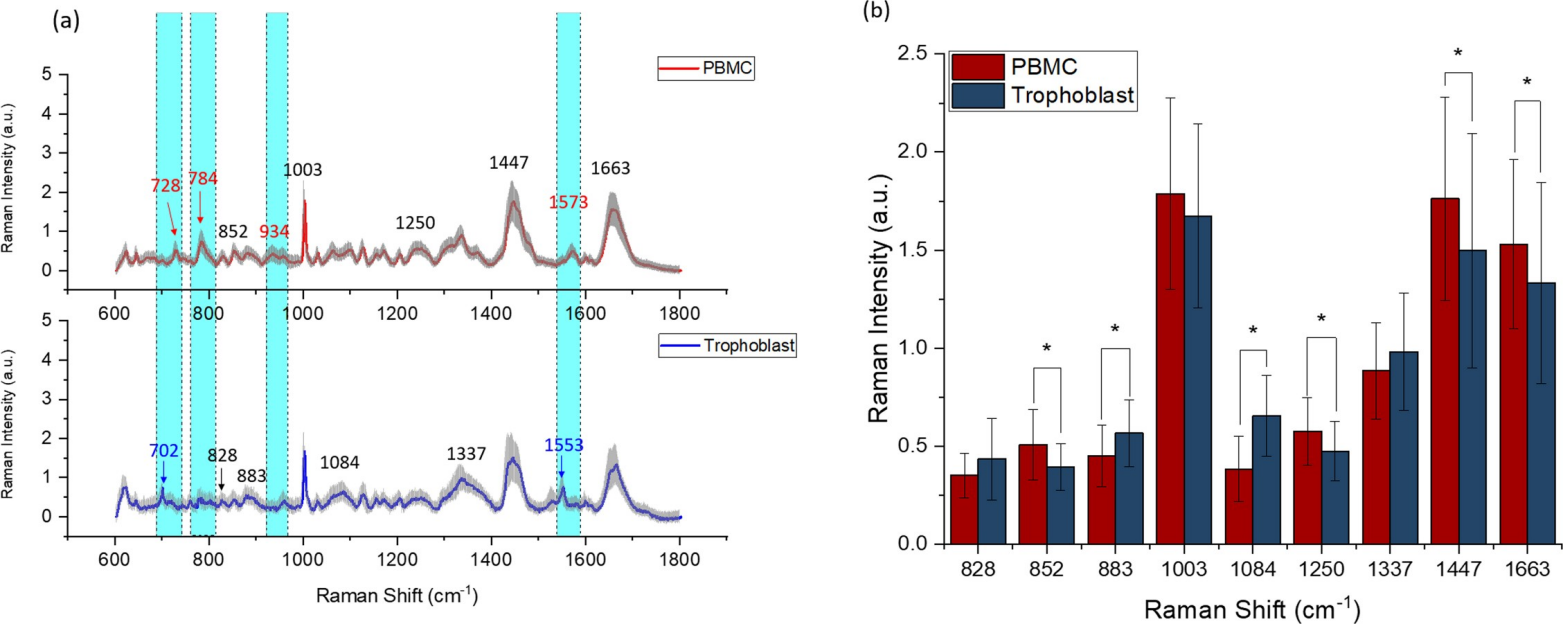
Raman fingerprint of extracellular vesicles (EVs) isolated from peripheral mononuclear blood cells (PBMC) and trophoblast cells. The solid line indicates the average of 90 spectra ± 1 standard deviation (shaded grey areas). The areas highlighted in cyan color showed the intensity differences. The peaks marked in red only exist in PBMC-derived EVs, while the peaks in blue only exist in trophoblast-derived EVs (a). Mean Raman peak intensity analysis of PBMC and trophoblast-derived EVs, *N = 90*, * means P < 0.05 (b).

After spectra collection, the baselines were subtracted before analysis using the software WiRE 3.4. Fig 5A illustrates the mean spectra comparison of the two types of EVs. The spectra showed characteristic bands of nucleic acids (720–820 cm^-1^), phenylalanine (1002 cm^-1^), lipid and protein markers indicated by the CH and CH_2_ groups (bands centered at 1450 cm^-1^).[26] In particular, lipids made a large contribution, which is in line with previously reported spectroscopic evidence.[19, 27–29] The differences of lipid cargo indicate that EVs derived from PBMCs and trophoblast cells may have distinct regulatory functions of other cell types since these lipids are involved in EV-cell interactions and signaling to cell membranes. The related assignments of spectral peaks to biological samples and chemical bonds are listed in S2 Table. The comparison of the average spectra revealed several spectral differences between the two EV types. The major differences in terms of peak location are highlighted in cyan color in Fig 5A. Peaks including 727/8 cm^-1^ (collagen assignment), 934/5 cm^-1^ (C-C backbone/C-C stretching mode of proline/ protein backbone), and 1573 cm^-1^ (purine/protein) were detectable only in PBMC spectra. Peak 784 cm^-1^ (DNA/RNA) was significantly higher in PBMC-derived EVs spectra than that in trophoblast-derived EVs. Peaks including 702 cm^-1^ (cholesterol) and 1553 cm^-1^ (tryptophan/protein assignment) were only found in trophoblast-derived EV spectra. These peaks could be used as “spectral markers” for the EV characterization, and therefore, contribute to the development of studies to evaluate the potential clinical application of this technology. In addition, Raman peak intensity analysis of six “spectral markers” (peaks 702, 728, 784, 934, 1553, 1573 cm^-1^) for animals are shown in S3 Fig. The counts of trophoblast and PBMC-derived EVs are significantly different for all six peak locations, which indicates that these “spectral markers” that can be used even at the single animal level. Other soluble factors outside extracellular vesicles may be present in the ultracentrifugation pellet. However, the previous centrifugation steps promote the enrichment of EVs in the final preparation and the remarkable peaks are most likely related to EV content.

Fig 5B compares the mean spectra of PBMC- and trophoblast-derived EVs on several characteristic peaks (828 cm^-1^, 852 cm^-1^, 883 cm^-1^, 1003 cm^-1^, 1084 cm^-1^, 1250 cm^-1^, 1337 cm^-^, ^1^1447 cm^-1^ and 1663 cm^-1^) which are indicated with black numbers in Fig 5A. The overall intensity of peaks such as 852 cm^-1^(glycogen), 1003 cm^-1^ (phenylalanine), 1250 cm^-1^ (amide III), 1447 cm^-1^ (CH_2_ bending mode of proteins & lipids) and 1663 cm^-1^ (proteins, including collagen I) was greater in PBMC-derived EVs.

Peripheral mononuclear blood cells in our samples are mainly composed of lymphocytes (70 to 90%) and monocytes (5 to 20%). Monocytes are the precursors for macrophages and the latter have tissue repair as one of their main functions. Cells associated with tissue repair produce or induce the production of collagen. Macrophages produce collagen into the extracellular matrixes to promote tissue stabilization.[30] Moreover, vesicles from different cell types associated with tissue healing carry collagen.[31] Therefore, macrophage-derived EVs can be the reason why peak 1663 cm^-1^ (proteins, including collagen I) was higher in the PBMC EV spectra. In addition, these results imply that EVs from PBMC have higher protein content than vesicles from trophoblast cells. Conversely, trophoblast-derived EVs have more protein and nucleic acid in its encapsulation as seen by their greater intensity at peaks 828 cm^-1^ (DNA/RNA), 883 cm^-1^ (protein), 1084 cm^-1^ (phosphodiester groups in nucleic acids) and 1337 cm^-1^ (protein and DNA). These data are helpful to determine how to further analyse these vesicles. Proteomics may be more suitable for PBMC-derived EVs than for trophoblast-derived EVs, while transcriptome analyses can be more relevant for the study of trophoblast-derived EVs. The substantial contribution of lipids for the Raman signals encourages us to follow with functional studies, since these vesicles probably interact differently with other cells, stimulating distinct metabolic pathways.

### 3.4 Principal component analysis and linear discriminant analysis

**Fig 6:**
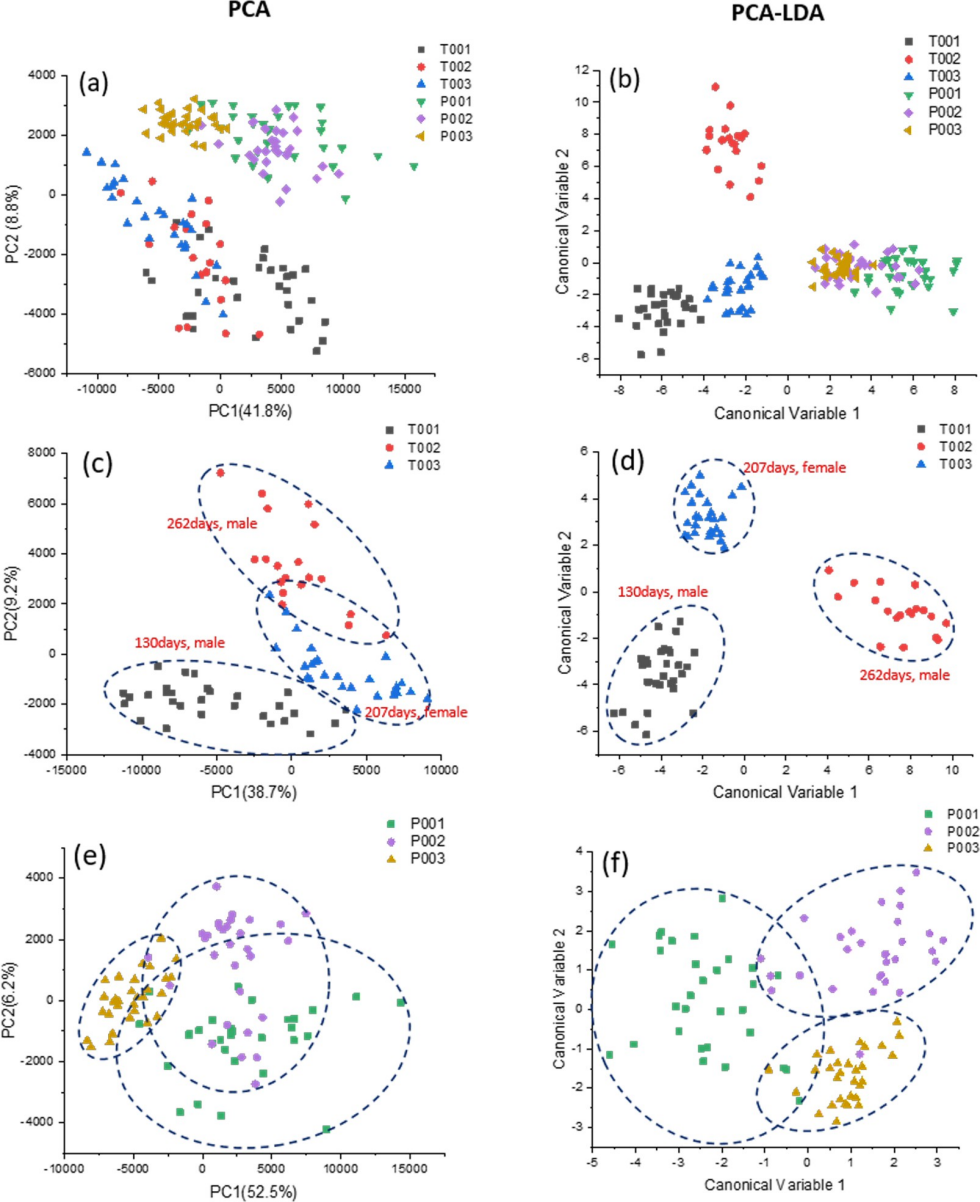
PCA and LDA analyses of the extracellular vesicles derived from peripheral mononuclear blood cells (P001, P002 and P003) and trophoblast cells (T001, T002 and T003). Scatter plot of the PCA results show the PC1 and PC2 scores assigned to each spectrum (*N = 90*) (a). Linear discriminant analysis, the first 10 PC loadings calculated by means of PCA were used for LDA. Each spot represents a single spectrum. The crosses indicate the mean canonical observation score obtained for each group (*N = 90*) (b). 2D PCA (c) and LDA (d) plots of PBMC-derived vesicles from three different cows, and 2D PCA (e) and LDA (f) plots of trophoblast-derived vesicles from three different animal individuals.

Further analysis of the Raman spectra endorsed the fact that Raman Spectroscopy can clearly distinguish trophoblast-derived EVs from PBMC-derived EVs and it is a suitable technique to study these vesicles. PCA analysis is an unsupervised learning algorithm aiming to find the components that maximize the variance in the data (i.e., within-variable variance) to find the differences. PCA analysis was conducted over the baseline subtracted Raman spectra on the range of 600 to 1800 cm^-1^. The first ten principal components (PCs) in PCA that reached 70% of variance were used for LDA, which made possible to verify the ability of the method to identify between-group differences by maximizing the variance among groups while minimizing intra-group variability. Fig 6A exhibits a 2D PCA plot including PC1 and PC2 of the six animal samples used in this study. In these PCA score plots, the first and second principal components were found to incorporate 50.6% (Fig 6A) of the total variance, respectively. It was clearly seen that PCA can distinguish the mean spectral differences for the two types of vesicles (PMBC vs. trophoblast) in 2D (Fig 6A) PCA graphs. In contrast to PCA, LDA is used to identify the feature subspace (i.e., a linear combination of the observed variables) that maximize class separation (i.e. between-class variance). The 2D LDA scatter plot (Fig 6B) revealed that the spectra of trophoblast-derived EVs fell into a region that was clearly separated from those of PBMC-derived EVs. The capability of the Raman spectroscopy method followed by PCA to distinguish between trophoblast derived EVs and EVs found in blood represents the first step in optimizing this technique to assess placental development, hence pregnancy health. LDA scatter plots also distinguished trophoblast EVs collected from different animals (Fig 6B), which was not achieved by the PCA model (Fig 6A).

**Fig 7:**
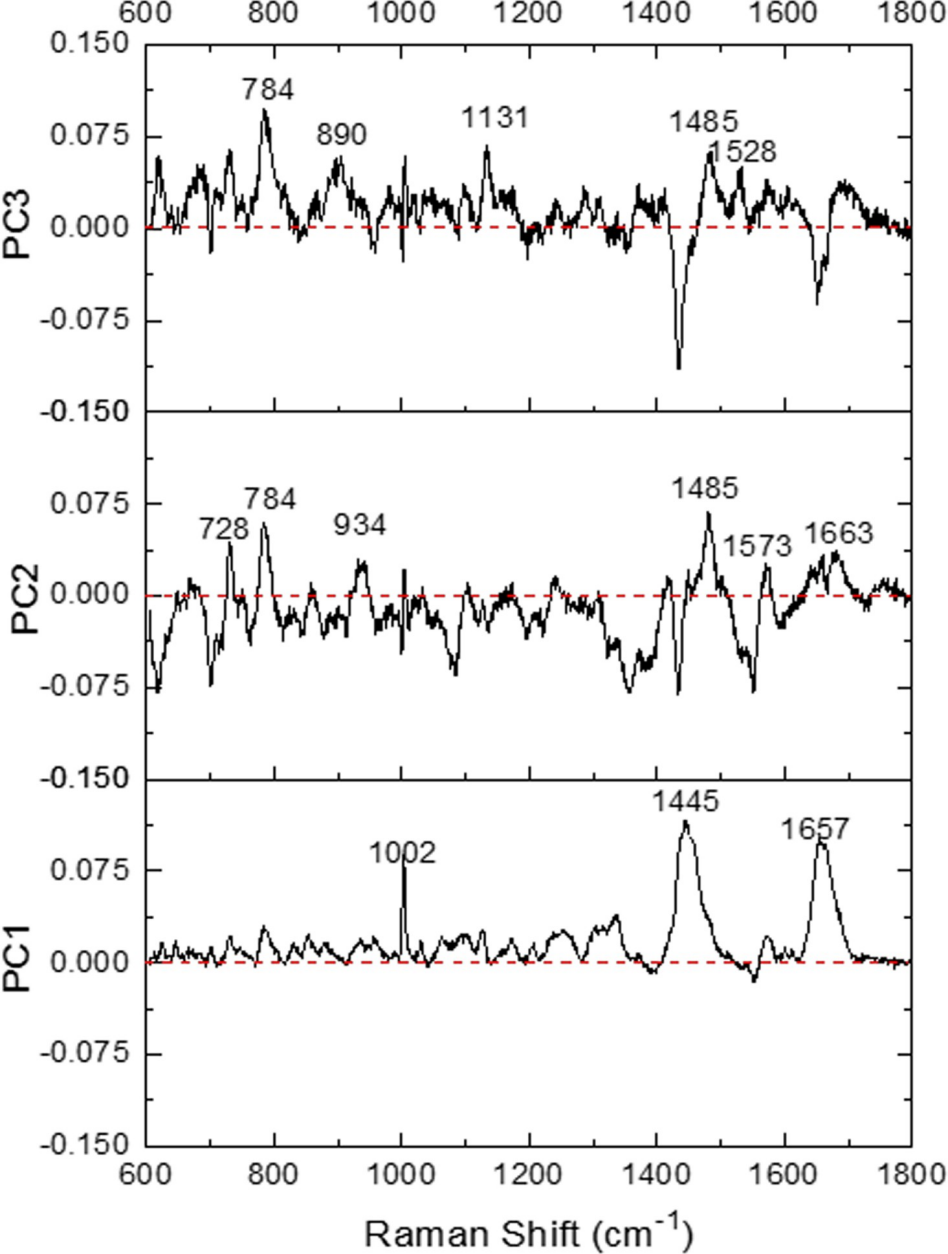
Principal component analysis (PCA) loading plots corresponding to PC1, PC2, and PC3 from the data of Fig 6A for extracellular vesicles derived from peripheral mononuclear blood cells and trophoblast cells.

As for the analysis of the data within the same type of EVs, the trophoblast group unexpectedly presents better separation regarding PCA (Fig 6C) or LDA plot (Fig 6D) than those in the PBMC group (Fig 6E and Fig 6F). T001 can be mostly separated from T002 and T003 by PCA (Fig 6C, S4A Fig), and all three individuals in this group are better distinguished from each other by LDA (Fig 6D). These results indicate that the biochemical composition of these two different types of EVs may be significantly different among these three animals. However, EVs isolated from PBMCs of three different animals are difficult to be discriminated at 2D (Fig 6E) and 3D (S4B Fig) PCA and were only partially separated in 2D LDA (Fig 6F), which means that the components of PBMC-derived EVs are similar regardless of the heterogeneity of mononuclear cell types in the sample and screening sample variation method used. In addition, PCA loading plots (Fig 7) from the data in Fig 6A show that classification might be majorly contributed by peaks at 1002 cm^-1^ (phenylalanine), 1445 cm^-1^ (proteins & lipids) and 1657 cm^-1^ (fatty acid/collagen) for PC1. As for PC2 and PC3, the main peaks contributing to the discrimination are 728 cm^-1^ (collagen), 784 cm^-1^ (DNA/RNA), 934 cm^-1^ (protein band), 1485 cm^-1^ (Amide II/nucleotide acid purine bases), 1573 cm^-1^ (guanine, adenine, TRP (protein)) for PC2, and 1663 cm^-1^ (DNA), and peaks at 784 cm^-1^, 890 cm^-1^ (protein), 1131 cm^-1^ (fatty acid), 1485 cm^-1^ and 1528 cm^-1^ (carotenoid) for PC3.

**Fig 8:**
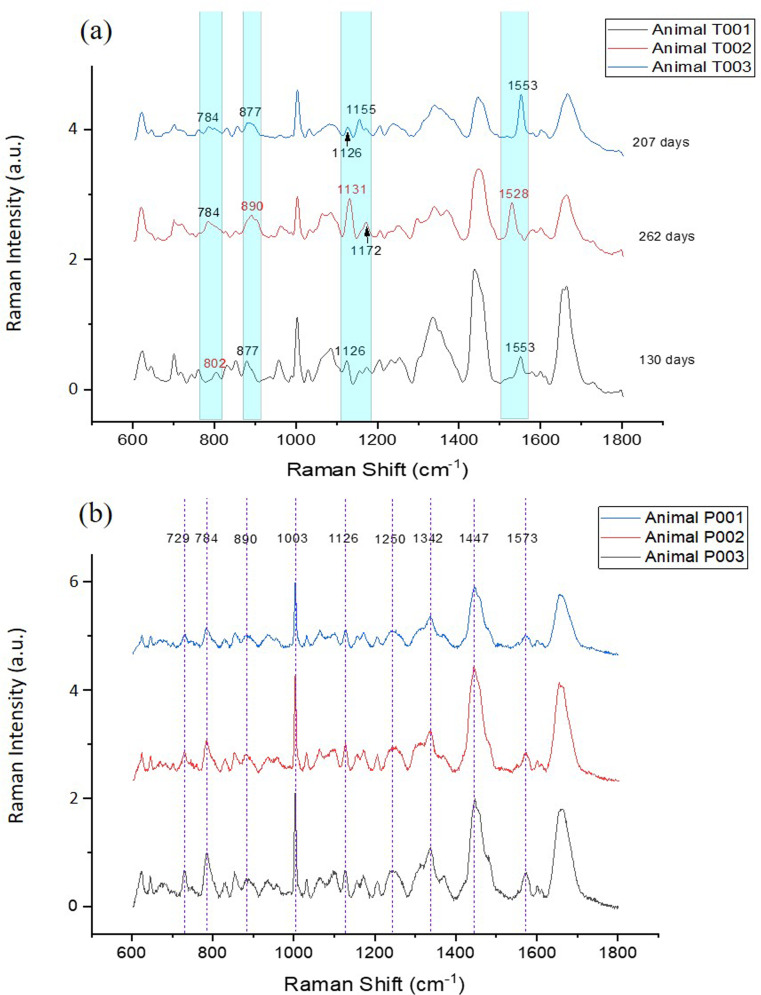
Raman fingerprints of extracellular vesicles. Raman spectra of trophoblast (a) and peripheral mononuclear blood cell (PBMC) (b) extracellular vesicles, *n = 30–35*. The spectral shift positions are highlighted in cyan color and indicated by red numbers.

To investigate the characteristic peaks related to each type of EV, the mean spectra of trophoblast vesicles from each animal are illustrated in Fig 8A. The overall spectral intensity of animal T001 was stronger than those of animals T002 and T003. This could be one of the main reasons why animal T001 was almost separated in the 2D PCA plot from T002 and T003 (Fig 6C). Besides an intensity difference, a peak shift was also observed in the mean spectra among animals T001, T002 and T003 (Fig 8A). In animal T001, peak 784 cm^-1^ (phosphodiester; cytosine/DNA and RNA) was shifted to 802 cm^-1^ (uracil-based ring breathing mode). In animal T002, peak 877 cm^-1^ (lipid) was shifted to 890 cm^-1^ (protein bands), peak 1126 cm^-1^ (lipid and protein) shifted to 1131 cm^-1^ (fatty acid), and peak 1553 cm^-1^ (tryptophan/amide II) shifted to 1528 cm^-1^ (carotenoid). Moreover, peak 1155 cm^-1^ (protein/glycogen)) and peak 1172 cm^-1^ (tyrosine) are absent in animal T002 and T003, respectively. The peak shift from 1553 cm^-1^ to 1528 cm^-1^ in animal T002 could also explain the lack of statistical significance between T002 and all the PBMC-derived EV samples (P001, P002 and P003; S3E Fig). Different from trophoblast-derived EVs, the PBMC-derived EVs from all three animals were found to only have intensity differences on peak counts. No peak shifts or distinctive peaks were observed (Fig 8B). This result, to some extent, suggests that impurities might be present in isolated EV samples, but they are not detectable in our Raman measurement. When specific antibodies are available in the future, a more specific evaluation of the presence of possible impurities could be confirmed.

The separation of sample T001 from T002 and T003 in the 2D PCA plot suggests these samples contain different biochemical fingerprints (Fig 6C). This can be explained by the fact that T001 is composed by trophoblast-derived EVs from a second-trimester pregnancy (130 days of gestation), while T002 and T003 EVs were derived from third-trimester pregnancies (262 and 207 days of gestation, respectively; S1 Table). While there is a number of studies investigating the role of EVs in pregnancy in humans, little is known about if and how EV content changes during gestation. It is well reported that EVs contain lipids, RNAs (including messenger RNA and microRNA), DNA and proteins in multiple species.[32] Trophoblast EVs have been demonstrated to promote maternal immunomodulation[33–35] and endothelial cell migration,[36] and the function of these vesicles changes over time. For instance, EVs derived from first-trimester human trophoblast cells have been shown to have different inflammatory properties compared to trophoblast EVs from pregnancies at later stages of gestation. [37] Therefore, since trophoblast cells and EVs at different stages of gestation exert distinct functions, it is reasonable to expect that the trophoblast EV cargo will change over the course of a pregnancy. The concentration of exosomes has been shown to increases in maternal circulation during pregnancy by 20-fold compared to non-pregnant women.[4] Additionally, the number of exosomes released by the placenta increases as pregnancy progresses. In this study, we analysed the biochemical fingerprints of EVs in general, which refer to a combination of exosomes, microvesicles and apoptotic bodies. The difference in Raman spectra among T001 and T002 and T003 could also be ascribed by the fact that the composition (proportion of exosomes to microvesicles and apoptotic bodies) of the EV pool changes as pregnancy progresses; therefore, skewing the Raman spectra results.

## 4 Conclusion

In this study, Raman spectroscopy was employed to investigate the differences in biochemical composition between bovine trophoblast and PBMC-derived EVs for the first time. Certain peaks only appeared in PBMC EVs, while others only existed in trophoblast-derived EVs. This finding can be further exploited and potentially could be used as the criteria for classification to differentiate bovine EVs from distinct sources without the use of any labeling system. Here we demonstrated that Raman spectroscopy is not only an effective method to detect specific biochemical fingerprints between bovine trophoblast and PBMC derived EVs, but also to potentially distinguish trophoblast-derived EVs from different stages of pregnancy using small sample volumes. Since trophoblast EVs are present in the maternal circulation during gestation, this technique has the potential to be applied as a tool to non-invasively monitor placental development and to diagnose placenta-associated pregnancy complications. Moreover, this work will aid in the design of functional studies as well as a more in-depth characterization of trophoblast-derived EVs. However, a more study should be performed with larger groups of samples to verify and optimize the use of Raman spectroscopy for this application.

## Supporting information

S1 Fig Pellet recovered after ultracentrifugation of trophoblast cell culture supernatant. (TIF)

S2 Fig Raman spectra comparison. MgF_2_ substrate (black), phosphate buffered solution (PBS) buffer on MgF_2_ substrate (red), and extracellular vesicles (EV) derived from trophoblast cells loaded on MgF_2_ (blue) under 50× magnification at 100% laser power, 10 seconds, exposure time and 1-time accumulation.
(TIF)

S3 Fig Raman peak intensity analysis of trophoblast and peripheral blood mononuclear cells. (PBMC) derived extracellular vesicles (EVs) at the “spectral markers” characteristic peaks: 702cm^-1^(a), 728cm^-1^ (b), 784cm^-1^ (c), 934cm^-1^ (d) 1553cm^-1^ (e) and 1573cm^-1^ (f), * *P* < 0.05.
(TIF)

S4 Fig 3D Principal component analysis (PCA) plots. PCA of trophoblast-derived extracellular vesicles from three different animals (T001, T002 and T003) (a), and peripheral blood mononuclear cell-derived vesicles from other three animals (P001, P002 and P003) (b).
(TIF)

S1 Table Cows used in the study. (DOCX)

S2 Table Relevant Raman peak assignments. (DOCX)

S1 Data (DOCX)
