# Supplementary figures and images for: Raman Spectroscopy characterization extracellular vesicles from bovine placenta and peripheral blood mononuclear cells
Source: PLoS One. 2020 Jul 2;15(7):e0235214. doi: 10.1371/journal.pone.0235214 (PMC7332028; doi:10.1371/journal.pone.0235214)

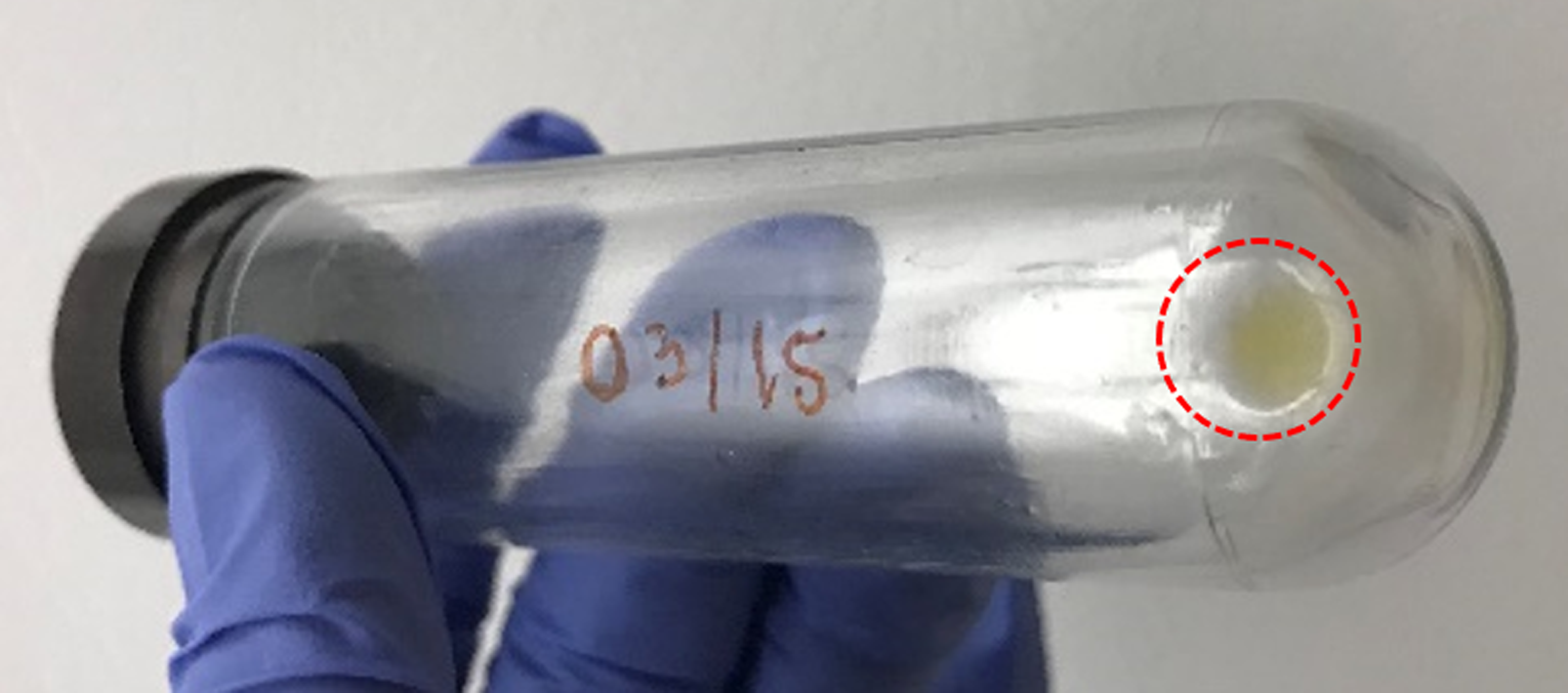

Supplement: S1 Fig — (TIF) [file pone.0235214.s001.tif]

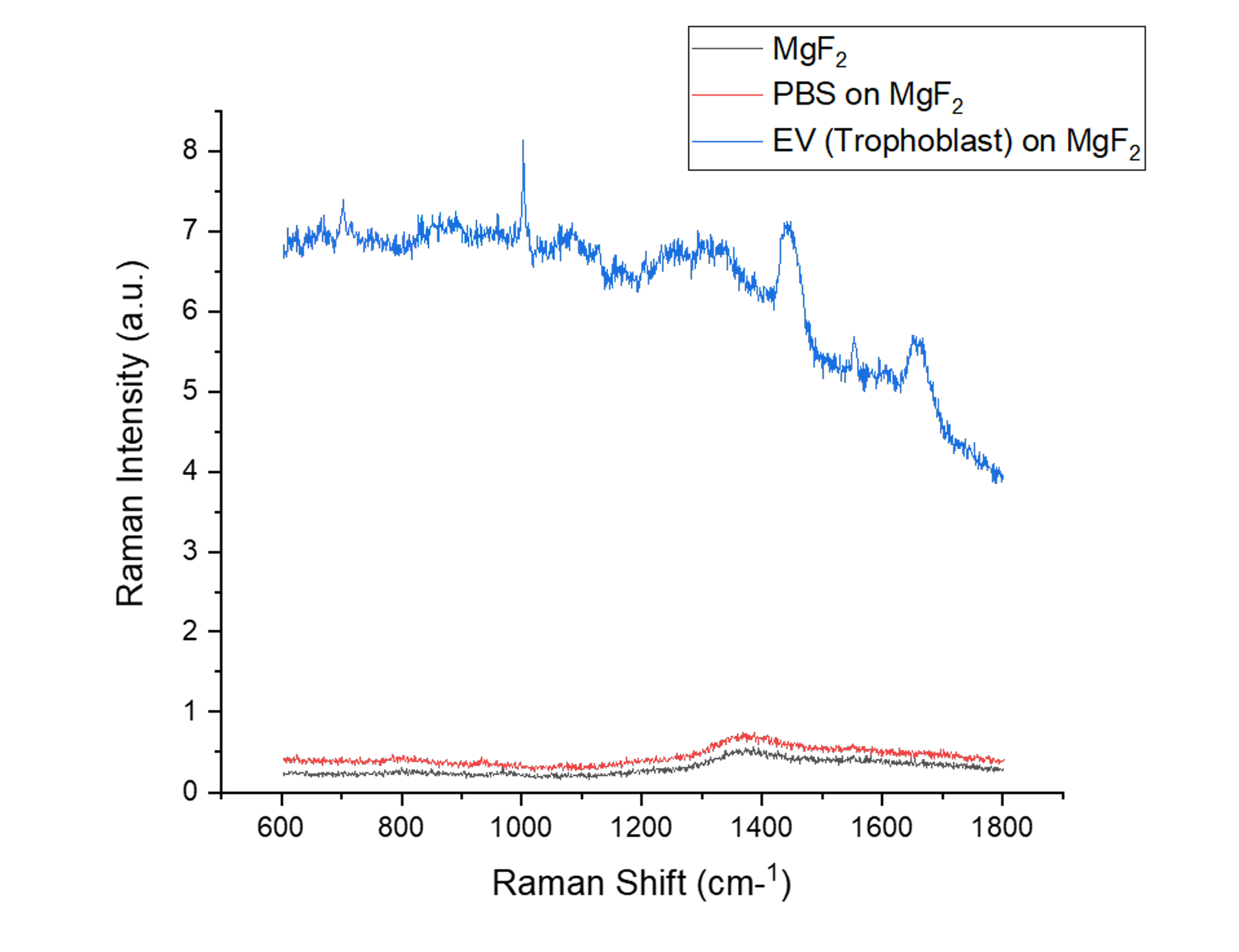

Supplement: S2 Fig — MgF2 substrate (black), phosphate buffered solution (PBS) buffer on MgF2 substrate (red), and extracellular vesicles (EV) derived from trophoblast cells loaded on MgF2 (blue) under 50× magnification at 100% laser power, 10 seconds, exposure time and 1-time accumulation. (TIF) [file pone.0235214.s002.tif]

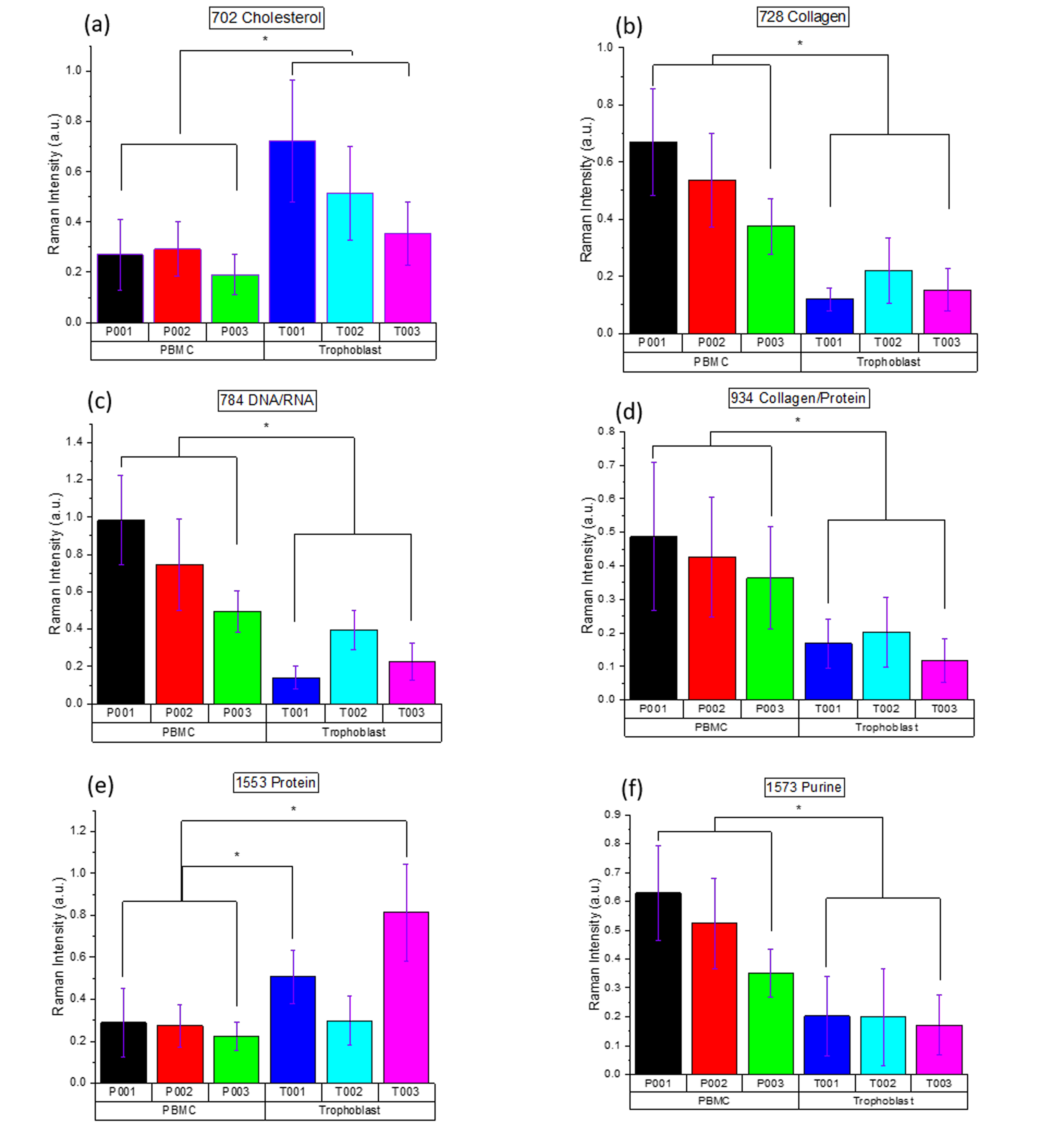

Supplement: S3 Fig — (PBMC) derived extracellular vesicles (EVs) at the “spectral markers” characteristic peaks: 702cm-1(a), 728cm-1 (b), 784cm-1 (c), 934cm-1 (d) 1553cm-1 (e) and 1573cm-1 (f), * P < 0.05. (TIF) [file pone.0235214.s003.tif]

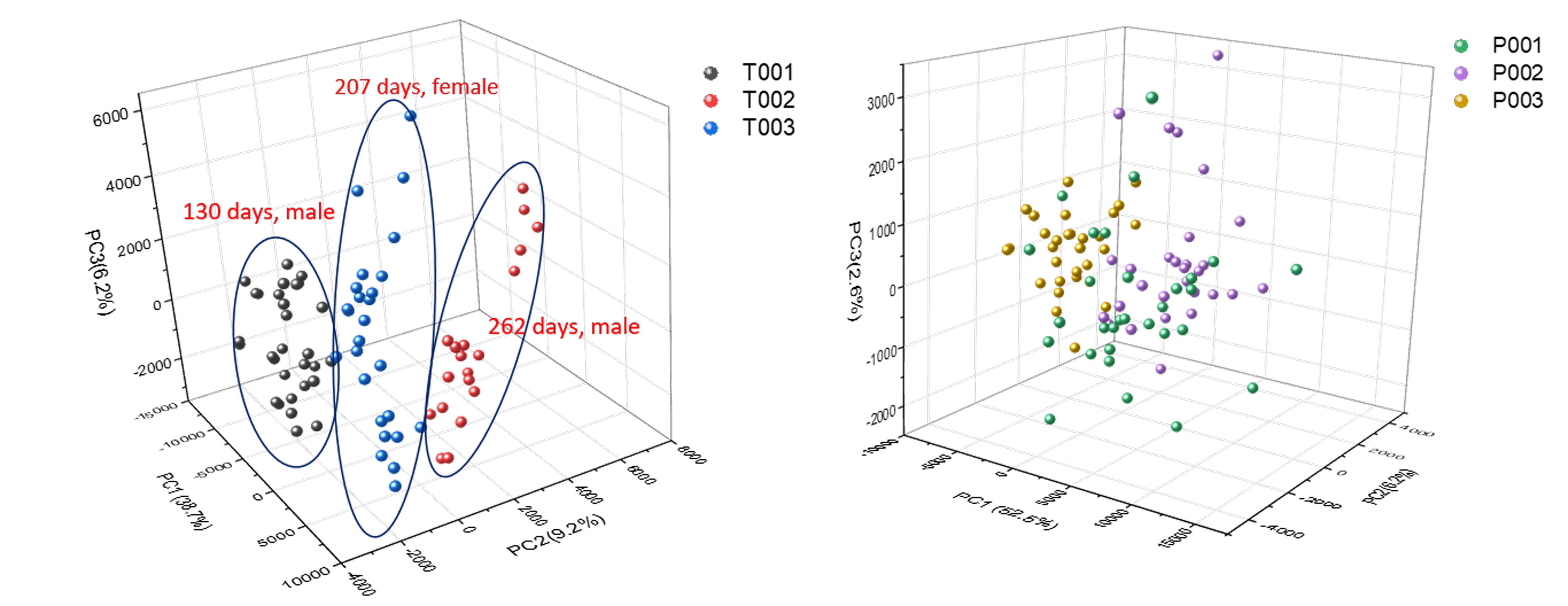

Supplement: S4 Fig — PCA of trophoblast-derived extracellular vesicles from three different animals (T001, T002 and T003) (a), and peripheral blood mononuclear cell-derived vesicles from other three animals (P001, P002 and P003) (b). (TIF) [file pone.0235214.s004.tif]
