# Supplementary material for: Raman Spectroscopy characterization extracellular vesicles from bovine placenta and peripheral blood mononuclear cells
Source: PLoS One. 2020 Jul 2;15(7):e0235214. doi: 10.1371/journal.pone.0235214 (PMC7332028; doi:10.1371/journal.pone.0235214)
Supplement: S1 Table — (DOCX) [file pone.0235214.s005.docx]

**S1 Table** **Cows used in the study.**

| Cow Number | Gestational status | Gestational age (days) | Fetal sex | Lactation Days |
| --- | --- | --- | --- | --- |
| P001 | No | N/A | N/A | 90 |
| P002 | No | N/A | N/A | 90 |
| P003 | No | N/A | N/A | 85 |
| T001 | Yes | 130 | male | N/A |
| T002 | Yes | 262 | male | N/A |
| T003 | Yes | 207 | female | N/A |
