# Supplementary material for: Raman Spectroscopy characterization extracellular vesicles from bovine placenta and peripheral blood mononuclear cells
Source: PLoS One. 2020 Jul 2;15(7):e0235214. doi: 10.1371/journal.pone.0235214 (PMC7332028; doi:10.1371/journal.pone.0235214)
Supplement: S2 Table — (DOCX) [file pone.0235214.s006.docx]

**S2 Table** **Relevant Raman peak assignments.**

| **Position (cm^−1^)** | **Assignment** |
| --- | --- |
| 702 | Cholesterol ester |
| 727/8 | C-C stretching, proline (collagen assignment) |
| 782-786 | DNA/RNA/Phosphodiester/cytosine |
| 802 | Uracil-based ring breathing mode |
| 828/30 | DNA/RNA |
| 852/3 | Glycogen |
| 880-884 | Protein |
| 890 | Protein |
| 934/5 | C-C backbone/C-C stretching (collagen/protein assignment) |
| 1003 | Phenylalanine |
| 1084 | Phosphodiester groups in nucleic acids |
| 1124/6 | n(C-C) skeletal of acyl backbone in lipid/C-N stretching vibration (protein vibration) |
| 1131 | Fatty acid |
| 1155 | Protein/ Glycogen |
| 1172 | tyrosine |
| 1250 | Amide III |
| 1337/9 | Protein and DNA |
| 1445/7 | Proteins & lipids assignment |
| 1485 | Amide II/nucleotide acid purine bases |
| 1528 | Carotenoid |
| 1552-1554 | Tryptophan (protein assignment)/porphyrin/Amide II |
| 1573 | Guanine, adenine, TRP (protein) |
| 1657 | Fatty acid/collagen |
| 1663 | DNA |

The assignment information in this table are based on the article published by Movasaghi et al[^4^](#_ENREF_4)
