# Supplementary material for: Raman Spectroscopy characterization extracellular vesicles from bovine placenta and peripheral blood mononuclear cells
Source: PLoS One. 2020 Jul 2;15(7):e0235214. doi: 10.1371/journal.pone.0235214 (PMC7332028; doi:10.1371/journal.pone.0235214)
Supplement: S1 Data — (DOCX) [file pone.0235214.s007.docx]

**Supplemental Materials**

Raman Spectroscopy Characterization of Extracellular Vesicles Derived from Bovine Placenta and Peripheral Blood Mononuclear Cells

Han Zhang^1¶^, Ana Caroline Silva^2¶^, Wei Zhang^1^, Heloisa.Rutigliano^2*^ Anhong Zhou^1*^

*^1^Department of Biological Engineering, Utah State University, Logan, Utah 84322-4105, USA*

*^2^Department of Animal Dairy and Veterinary Sciences, Utah State University, Logan, Utah 84322, USA*

* Corresponding authors

E-mail: [anhong.zhou@usu.edu](mailto:anhong.zhou@usu.edu); [heloisa.rutigliano@usu.edu](mailto:heloisa.rutigliano@usu.edu)

¶ These authors contributed equally to this work.

**Spectral data analysis Method**

Unsupervised methods are very useful to find hidden structures in the unlabelled data and are often used as a precursor to supervised methods when working on large data sets. [^1^](#_ENREF_1)

As an unsupervised method, PCA is one widely used dimensionality reduction technique. This technique is used for projecting a higher dimensional data matrix “X” onto a low component subspace. Its idea is simple—reduce the dimensionality of a dataset, while preserving as much ‘variability’ (i.e. statistical information) as possible,[^2^](#_ENREF_2) the formula is given below:

$$X=Y{U_{k}}^{T}+E=structure+Noicse$$

$$Y=XU_{k}$$

where X is the original m × n data matrix; Y is the m × k scores matrix and U_k_ are then n × k loadings matrix.

The principal component analysis is able to identify some important structural data information, but it has less discrimination power due to the fact that it is an unsupervised procedure.[^1^](#_ENREF_1) Often, interpretation of the complex biochemical information obtained through vibrational spectroscopic techniques requires further data analysis using supervised procedures such as linear discriminant analysis (LDA). This method tries to find the axes ‘W’ that maximizes the objective function (ratio of between-class scatter to within-class scatter) “J(W)” defined as below:

$$J\left( W \right)=\frac{|W^{T}SC_{b}W|}{|W^{T}SC_{w}W|}$$

where, SC_w_ means within-class scatter; SC_b_ means between class scatter; W= [w_1_ |w_2_ | ……… w_L_] and L is the number of solutions (projections).

The solution to this optimization problem is given by solving the generalized eigenvalue problem as shown below.

SC_b_w_i_− λ_i_SC_w_w_i_ = 0; i = 1; 2; 3; ………; L

where each w_i_ (eigenvector) gives a unique projection and λ_i_ is the corresponding eigenvalue.

Data reduction by PCA before LDA was essential since LDA requires the number of variables to be smaller than the number of observations.[^3^](#_ENREF_3) Principal component analysis is first applied to the original data set “X” and only the first few principal component scores are retained for further analysis. Therefore, the resulting principal component scores matrix “Y” is of size “m × k”. LDA is then applied on matrix “Y” to obtain the LD score Matrix “Z” as below.

Z = Y *W;

**S1 Table** **Cows used in the study.**

| Cow Number | Gestational status | Gestational age (days) | Fetal sex | Lactation Days |
| --- | --- | --- | --- | --- |
| P001 | No | N/A | N/A | 90 |
| P002 | No | N/A | N/A | 90 |
| P003 | No | N/A | N/A | 85 |
| T001 | Yes | 130 | male | N/A |
| T002 | Yes | 262 | male | N/A |
| T003 | Yes | 207 | female | N/A |


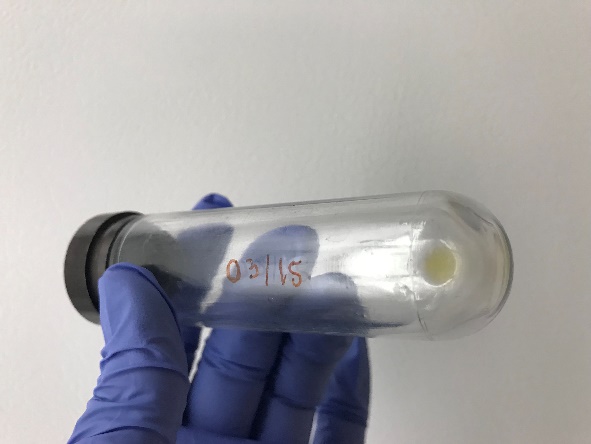


**S1 Fig. Pellet recovered after ultracentrifugation of trophoblast cell culture supernatant.**

**S2 Table** **Relevant Raman peak assignments.**

| **Position (cm^−1^)** | **Assignment** |
| --- | --- |
| 702 | Cholesterol ester |
| 727/8 | C-C stretching, proline (collagen assignment) |
| 782-786 | DNA/RNA/Phosphodiester/cytosine |
| 802 | Uracil-based ring breathing mode |
| 828/30 | DNA/RNA |
| 852/3 | Glycogen |
| 880-884 | Protein |
| 890 | Protein |
| 934/5 | C-C backbone/C-C stretching (collagen/protein assignment) |
| 1003 | Phenylalanine |
| 1084 | Phosphodiester groups in nucleic acids |
| 1124/6 | n(C-C) skeletal of acyl backbone in lipid/C-N stretching vibration (protein vibration) |
| 1131 | Fatty acid |
| 1155 | Protein/ Glycogen |
| 1172 | tyrosine |
| 1250 | Amide III |
| 1337/9 | Protein and DNA |
| 1445/7 | Proteins & lipids assignment |
| 1485 | Amide II/nucleotide acid purine bases |
| 1528 | Carotenoid |
| 1552-1554 | Tryptophan (protein assignment)/porphyrin/Amide II |
| 1573 | Guanine, adenine, TRP (protein) |
| 1657 | Fatty acid/collagen |
| 1663 | DNA |

The assignment information in this table are based on the article published by Movasaghi et al[^4^](#_ENREF_4)


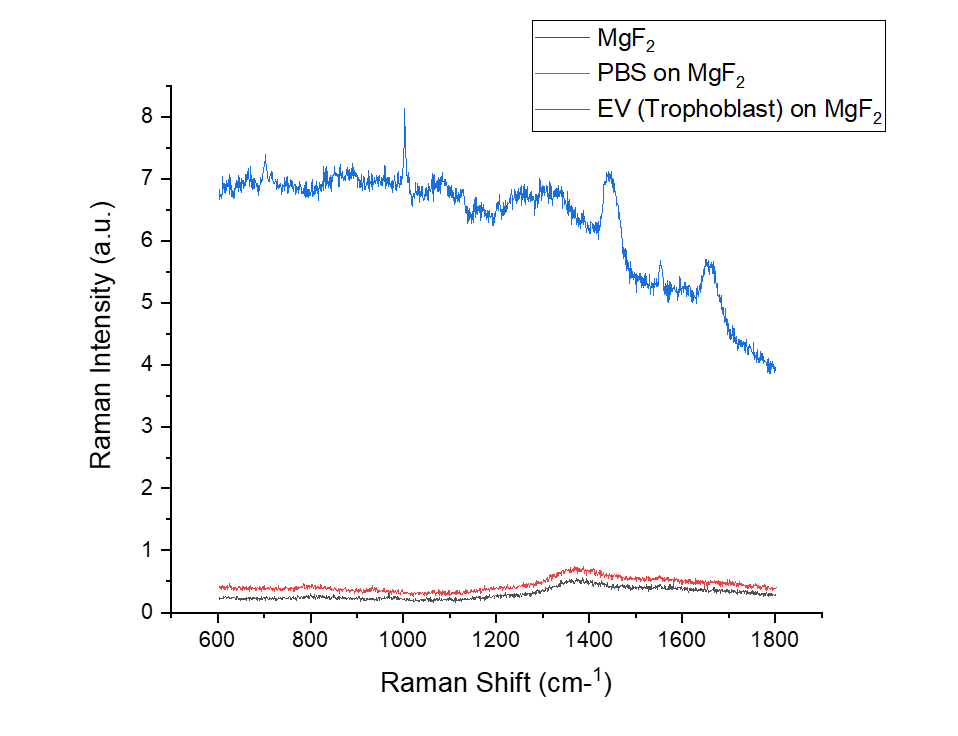


**S2 Fig.**  **Raman spectra comparison**. MgF_2_ substrate (black), phosphate buffered solution (PBS) buffer on MgF_2_ substrate (red), and extracellular vesicles (EV) derived from trophoblast cells loaded on MgF_2_ (blue) under 50× magnification at 100% laser power, 10 seconds, exposure time and 1-time accumulation.


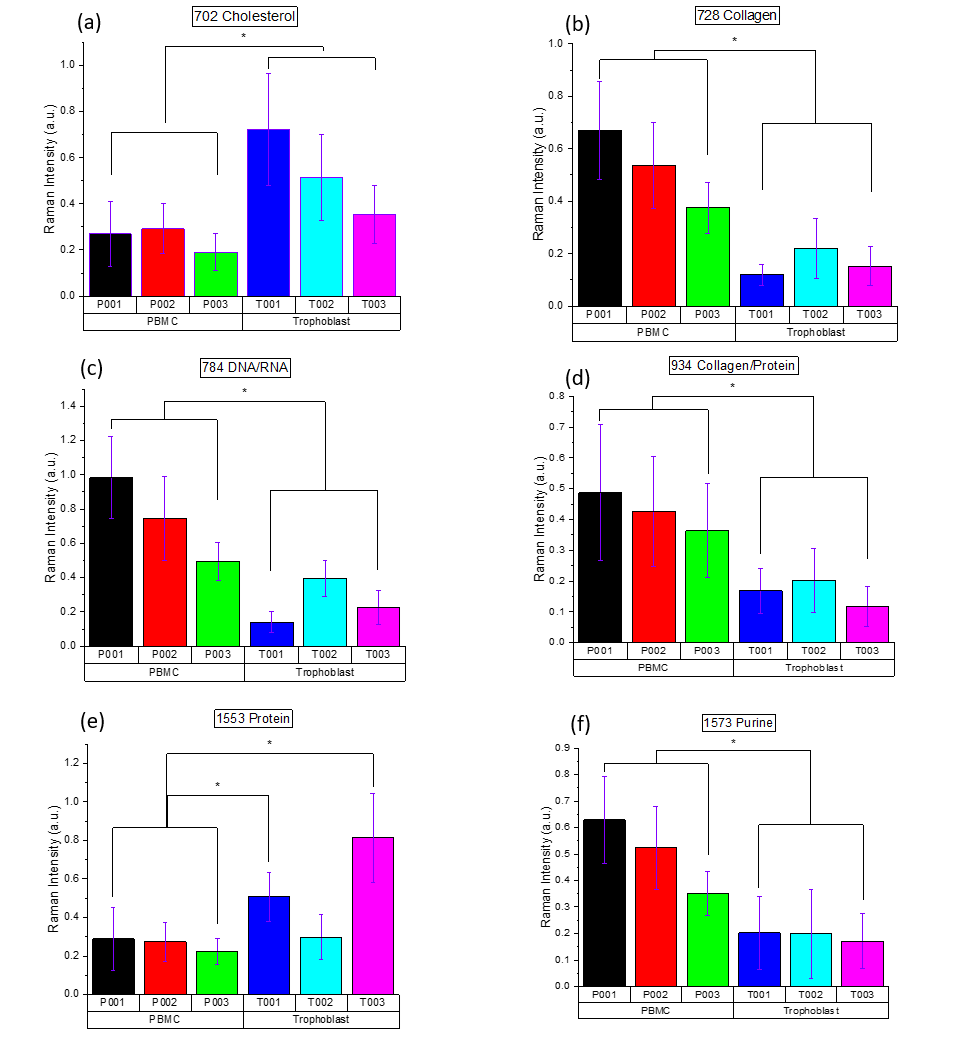


**S3 Fig. Raman peak intensity analysis of trophoblast and peripheral blood mononuclear cells.** (PBMC) derived extracellular vesicles (EVs) at the “spectral markers” characteristic peaks: 702cm^-1^(a), 728cm^-1^ (b), 784cm^-1^ (c), 934cm^-1^ (d) 1553cm^-1^ (e) and 1573cm^-1^ (f), * *P* < 0.05.

(a)

(b)

262 days, male

130 days, male

207 days, female

**S4 Fig. 3D Principal component analysis (PCA) plots.** PCA of trophoblast-derived extracellular vesicles from three different animals (T001, T002 and T003) (a), and peripheral blood mononuclear cell-derived vesicles from other three animals (P001, P002 and P003) (b).

**References**

1. R. Gautam, S. Vanga, F. Ariese and S. Umapathy, *EPJ Techniques and Instrumentation*, 2015, **2**, 8.

2. I. T. Jolliffe and J. Cadima, *Philos Trans A Math Phys Eng Sci*, 2016, **374**, 20150202.

3. N. T. Trendafilov and T. G. Gebru, *METRON*, 2016, **74**, 207-221.

4. Z. Movasaghi, S. Rehman and I. U. Rehman, *Applied Spectroscopy Reviews*, 2007, **42**, 493-541.
